# Evaluation of Aspirin Use With Cancer Incidence and Survival Among Older Adults in the Prostate, Lung, Colorectal, and Ovarian Cancer Screening Trial

**DOI:** 10.1001/jamanetworkopen.2020.32072

**Published:** 2021-01-15

**Authors:** Holli A. Loomans-Kropp, Paul Pinsky, Asad Umar

**Affiliations:** 1 Cancer Prevention Fellowship Program, Division of Cancer Prevention, National Cancer Institute, Rockville, Maryland; 2 Gastrointestinal and Other Cancers Research Group, Division of Cancer Prevention, National Cancer Institute, Rockville, Maryland; 3 Early Detection Research Branch, Division of Cancer Prevention, National Cancer Institute, Rockville, Maryland

## Abstract

**Importance:**

Many studies have evaluated the long-term benefits of aspirin use; however, the association of aspirin use with cancer incidence and survival in older individuals remains uncertain. Additional population-based evidence of this association is necessary to better understand any possible protective effects of aspirin in older adults.

**Objective:**

To investigate the association of aspirin use with risk of developing new cancers and site-specific cancer-associated survival in bladder, breast, esophageal, gastric, pancreatic, and uterine cancers.

**Design, Setting, and Participants:**

This cohort study used data from the Prostate, Lung, Colorectal, and Ovarian Cancer Screening Trial. Participants were aged 65 years or older at baseline (1993-2001) or reached age 65 during follow-up. Data analysis was conducted from January to June 2020.

**Main Outcomes and Measures:**

Incidence of and survival from the investigated cancer types. Univariable and multivariable hazard ratios (HRs) and 95% CIs were calculated using Cox proportional hazards regression modeling, adjusting for covariates. Multivariable models for incidence included time-varying covariates.

**Results:**

A total of 139 896 individuals (mean [SD] age at baseline, 66.4 [2.4] years; 71 884 [51.4%] women; 123 824 [88.5%] non-Hispanic White individuals) were included in the analysis. During the study period, 32 580 incident cancers (1751 [5.4%] bladder, 4552 [14.0%] breast, 332 [1.0%] esophageal, 397 [1.2%] gastric, 878 [2.7%] pancreatic, and 716 [2.2%] uterine cancers) were reported. Aspirin use was not associated with incidence of any of the investigated cancer types among individuals aged 65 years or older. Multivariable regression analysis demonstrated that aspirin use at least 3 times/week was associated with increased survival among patients with bladder (HR, 0.67; 95% CI, 0.51-0.88) and breast (HR, 0.75; 95% CI, 0.59-0.96) cancers but not among those with esophageal, gastric, pancreatic, or uterine cancer. A similar association of any aspirin use with bladder (HR, 0.75; 95% CI, 0.58-0.98) and breast (HR, 0.79; 95% CI, 0.63-0.99) cancer survival was observed.

**Conclusions and Relevance:**

In the current study, any aspirin use and aspirin use at least 3 times/week was associated with improved bladder and breast cancer survival. Associations between aspirin use and incidence of any of the investigated cancers or between aspirin use and esophageal, gastric, pancreatic, or uterine cancer survival were not observed.

## Key Points

**Question:**

Is aspirin use associated with incidence of or survival from breast, bladder, esophageal, gastric, pancreatic, or uterine cancer?

**Findings:**

In this cohort study of 139 896 participants from the Prostate, Lung, Colorectal, and Ovarian Cancer Screening Trial, aspirin use was not associated with reduced risk of breast, bladder, esophageal, gastric, pancreatic, or uterine cancers. However, it was associated with increased bladder and breast cancer survival.

**Meaning:**

These findings suggest that aspirin use may improve bladder and breast cancer survival.

## Teaser

This cohort study investigates the association of aspirin use with risk of developing new cancers and site-specific cancer-associated survival in bladder, breast, esophageal, gastric, pancreatic, and uterine cancers.

## Introduction

Aspirin is recommended for primary prevention of cardiovascular disease in individuals aged 50 to 59 years with high risk for that disease, with the additional stated benefit of a reduced risk of colorectal cancer.^1^ However, for those aged 60 to 69 years, recommendations are based on personalized risk-benefit profiles, and there is limited data for individuals younger than 50 years or older than 70 years. Despite potential risks, between 25% and 50% of adults in the United States have reported taking aspirin daily or every other day, with usage increasing with age.^2,3^ Long-term aspirin use has been associated with decreased risk of heart disease, stroke, cancer (particularly gastrointestinal cancers), and all-cause mortality.^1,4,5,6,7^ Recent research suggests that aspirin use may offer protection against the development of and mortality from other cancer types as well.^4,7,8^ However, the benefits and harms of taking low-dose aspirin in older individuals is still debated, particularly in light of data from the Aspirin in Reducing Events in the Elderly (ASPREE) study that indicated increased cancer-associated mortality—but not cancer incidence—with long-term aspirin use in individuals aged 65 years or older who were not taking aspirin before study enrollment.^9,10^

Secondary analyses of randomized clinical trials have shown protective associations for several gastrointestinal cancers, but there is a paucity of data suggesting preventive benefits in older individuals.^11,12^ For example, studies in esophageal cancer have shown up to 70% reduced risk of progression from Barrett esophagus to esophageal adenocarcinoma with the use of aspirin or other nonsteroidal anti-inflammatory drugs (NSAIDs), with increasing benefit with longer-term use, while cohort studies in China showed 38% to 58% reduced risk of gastric cancer with increased duration of aspirin use.^13,14,15,16,17,18^ Similar associations have been investigated in bladder, breast, pancreatic, and uterine (including uterine corpus endometrial carcinoma) cancers.^19,20,21,22,23^

Considering that aspirin use nonselectively inhibits cyclooxygenase, thus suppressing inflammation, it stands to reason that long-term aspirin use may affect cancer initiation.^24^ Recent large-scale evaluations have indicated aberrant immune cell activity in several tumor types, including bladder, breast, gastric, and uterine tumors, providing a prime target for aspirin activity.^25,26,27,28,29^ Furthermore, aspirin use has been demonstrated to affect survival after a cancer diagnosis, although the data remain mixed.^30,31^ We previously demonstrated that aspirin use 3 or more times per week was associated with reduced risk of cancer mortality, with additional studies demonstrating a similar inverse association between aspirin use and overall cancer mortality; however, the research investigating the association of aspirin use with cancer site–specific mortality in older individuals is limited.^9,32,33^

Due to the recent controversy surrounding aspirin use in older adults (ie, aged ≥65 years), we decided to focus our investigation on older participants in the Prostate, Lung, Colorectal and Ovarian (PLCO) Cancer Screening Trial.^9^ Recent investigations in the PLCO population have demonstrated several associations between aspirin use and the risk of cancer incidence and survival. Among cancers screened as part of the trial protocol, aspirin use has been associated with significant reductions in risk of colorectal polyps and colorectal cancer; in contrast, there was modest to no association between aspirin use and prostate and ovarian incidence and survival.^33,34,35,36,37^ To our knowledge, no investigations into aspirin use and risk of bladder, breast, esophageal, gastric, pancreatic, or uterine cancers have been conducted in PLCO. With the high frequency of aspirin use, the substantial annual incidence of breast, bladder, and uterine cancers in the United States, and the noted association between aspirin use and gastrointestinal cancers (eg, esophageal, gastric, pancreatic), we chose to perform a thorough investigation of the association between aspirin use and cancer risk and survival of these cancers in PLCO. We hypothesized that aspirin use would be associated with reduced risk of cancer incidence and increased cancer survival.

## Methods

The current cohort study is a post hoc analysis of the PLCO Cancer Screening Trial. The PLCO trial was initially approved by the institutional review boards of all study sites (ie, University of Alabama at Birmingham, Georgetown University, University of Pittsburgh, Washington University in St Louis, University of Utah, University of Colorado, University of Minnesota, Pacific Health Research and Education Institute [Hawaii], the Henry Ford Health System [Detroit, Michigan], and Marshfield Clinic Research Foundation [Marshfield, Wisconsin]). All participants provided written informed consent for the original and ancillary studies. The present study did not seek additional approval because data use in ancillary studies was included in the initial consent and all data were deidentified. This study adhered to the Strengthening the Reporting of Observational Studies in Epidemiology (STROBE) reporting guideline.

### Study Design

The overall design of PLCO has been described elsewhere and is available online.^38,39,40,41^ Briefly, participants aged 55 to 74 years were enrolled and randomized to the intervention or control group at 10 screening centers from November 8, 1993, to July 2, 2001. Pertinent exclusion criteria for the current analysis are history of prostate, lung, colorectal, or ovarian cancer; undergoing treatment for cancer (except for basal or squamous cell skin carcinoma); current participation in another cancer screening or cancer primary prevention trial; men who had taken Proscar, Propecia, or finasteride in the 6 months prior to randomization; and women who had taken tamoxifen or raloxifene in the 6 months prior to randomization. The tamoxifen or raloxifene criteria was lifted in 1996. Individuals randomized to the intervention group received screening examinations for prostate, lung, colorectal, and ovarian cancers in designated study years. Participants assigned to the control group received usual care.

The current study cohort included individuals aged 65 years or older at baseline or who survived to at least age 65 years after enrollment, with a valid baseline questionnaire (BQ) with completed aspirin use information. A supplemental questionnaire (SQ) was distributed to PLCO study participants between 2006 and 2008; however, completion of the SQ was not required for study inclusion. The BQ and SQ are publicly available.^42,43^ All responses were self-reported. Relevant to the current study, the BQ asked, “During the last 12 months, have you regularly used aspirin or aspirin-containing products, such as Bayer, Bufferin, or Anacin (Please do not include aspirin-free products such as Tylenol or Panadol)?” The SQ asked, “During the last 12 months, about how often did you usually take aspirin (examples of aspirin include Bayer, Bufferin, Anacin, and baby aspirin)?” Two frequency categories were used for analysis. First, participants were categorized as aspirin users if the response on the BQ was yes or the response on the SQ was greater or equal to 1 time per month, depending on when the SQ was completed. Second, an aspirin use threshold of less than 3 times/week and at least 3 times/week, based on previous work, was established by collapsing response categories in the BQ and SQ to fit the appropriate categories.^33^

The original analysis of PLCO study data was completed after 13 years of follow-up or on December 31, 2009, whichever came first.^39^ Participants were reconsented in 2011 to either continue follow-up or refuse further follow-up. Mortality follow-up continued until time of death, December 31, 2015, for those who consented to continued follow-up, or the refusal date for those who refused consent to continued follow-up. Follow-up for incident cancers continued until death, December 31, 2014, for those who consented to continued follow-up, or the end of 2009 for those who refused consent.

### Cancer Incidence and Survival Analysis

**Figure 1.**
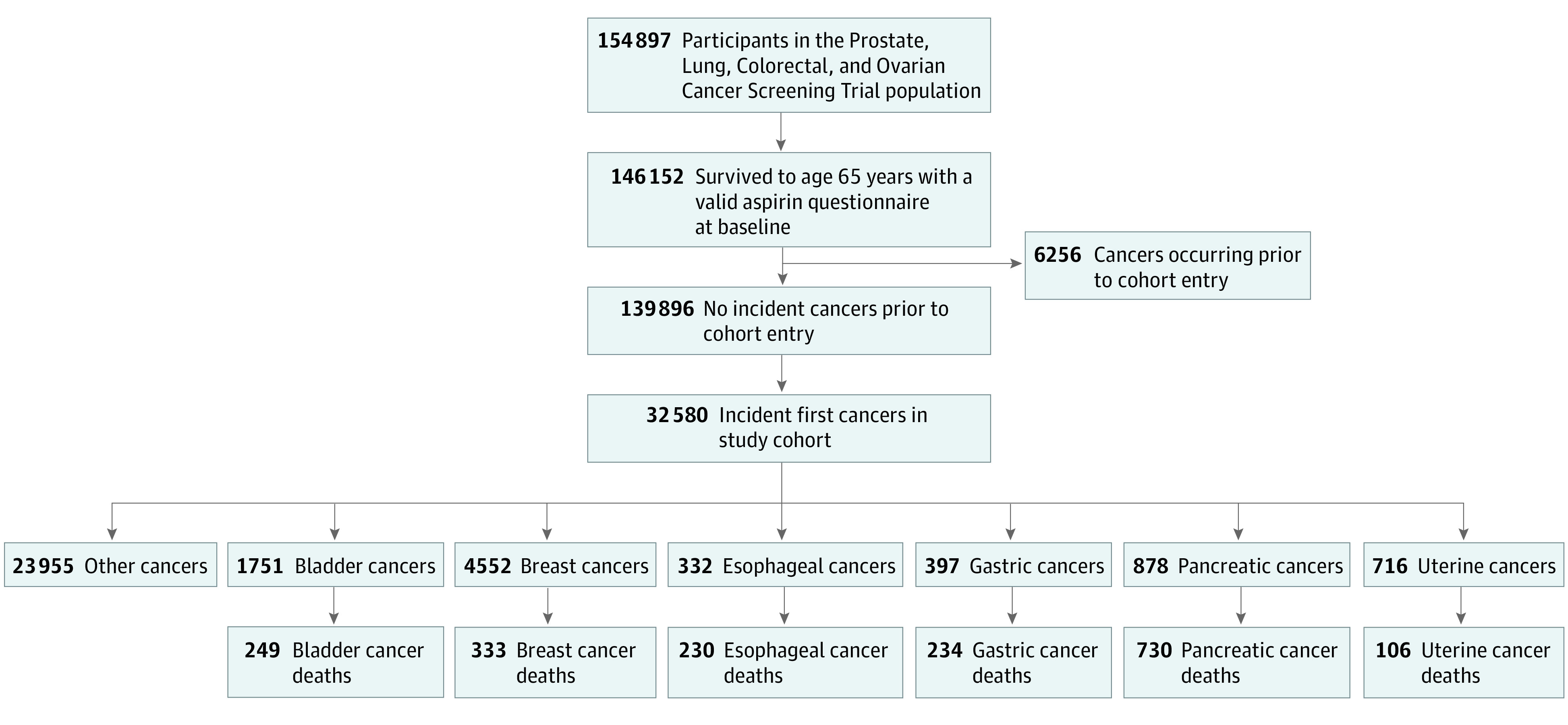
Flowchart of Eligible Prostate, Lung, Colorectal, and Ovarian Cancer Screening Trial Participants

The first goal of this analysis was to evaluate the association between aspirin use and incidence of bladder, breast, esophageal, gastric, pancreatic, and uterine (which included uterine corpus endometrial carcinomas) cancers among individuals aged 65 years and older in the PLCO Cancer Screening Trial (Figure 1). Incident cancers were defined as first cancers diagnosed during cohort follow-up. Follow-up time began at the time of randomization or when the participant reached age 65 years, whichever occurred first, and continued until the date of the cancer diagnosis, participant death, or the end of the study follow-up. The second goal was to examine the association of aspirin use prior to diagnosis with subsequent cancer-specific survival in participants with the previously listed incident cancers. Follow-up for this analysis began at the time of diagnosis and ended at death or end of study follow-up. Initially in PLCO, incident cancers and deaths were determined by annual updates, participant reports, medical record abstraction, and death certificates. Following reconsenting, incident cancers were determined by linkages to state cancer registries and deaths through linkages to the National Death Index.

The *International Classification of Diseases, Ninth Revision *(*ICD-9*) codes for incidence and reported causes of death for bladder (188.1, 188.9), breast (174.0, 174.9), esophageal (150.0, 150.5, 150.9), gastric (151.0, 151.9), pancreatic (157.0-157.4, 157.8, 157.9), and uterine (182.0) cancers were extracted for this analysis. Carcinoma in situ of the bladder and breast were included. Because there have been noted instances of misclassification of cause of death of esophageal and gastric cancers, if a participant with an incident esophageal or gastric cancer had a cause of death by gastric or esophageal cancer, respectively, those deaths were included in the survival analyses as events.^44,45^

### Statistical Analysis

Hazard ratios (HRs) were calculated using Cox proportional hazards regression models to assess the association between aspirin use and cancer incidence or survival, with the primary outcomes being any reported aspirin use (reference, no reported aspirin use) and reported aspirin use at least 3 times/week (reference, aspirin use <3 times/week).^33^ Additional covariates included in the regression models were study randomization group (intervention, control), sex (male, female), race (non-Hispanic White, non-Hispanic Black, other), smoking status (never, current, former), and history of heart attack, stroke, hypertension, and diabetes. Considering that the status of aspirin use as well as that of several covariates (eg, smoking status and history of heart attack, stroke, hypertension, and diabetes) may have changed between the completion of the BQ and SQ, time-dependent proportional hazards models for cancer incidence were used. If cohort entry preceded the SQ and the SQ was completed, aspirin and covariate data were taken from the BQ until the time of the SQ and from the SQ afterwards. If the SQ was completed prior to cohort entry, then only SQ data were used. For the survival analyses, time-independent proportional hazards models were used, with aspirin use and covariate data taken from the SQ if it was completed prior to diagnosis or otherwise from the BQ. Kaplan-Meier analyses of survival were also run, stratified by aspirin use categories (<3 times/week vs ≥3 times per week), with differences between groups assessed by the log-rank test. Participants who died of causes other than the cancer of interest were treated as censored at that time, both for the survival and proportional hazards analyses. All statistical analyses were performed using SAS version 9.4 (SAS Institute). A 2-tailed *P* < .05 was considered statistically significant. Data analysis was conducted from January to June 2020.

## Results

The eligible PLCO study population included 139 896 participants (mean [SD] age at baseline, 66.4 [2.4] years; 71 884 [51.4%] women; 123 824 [88.5%] non-Hispanic White individuals; 65 502 [46.8%] with no smoking history), summarized in the Table. In the current study, 32 580 incident cancers were reported, including 1751 (5.4%) bladder cancers, 4552 (14.0%) breast cancers, 332 (1.0%) esophageal cancers, 397 (1.2%) gastric cancers, 878 (2.7%) pancreatic cancers, and 716 (2.2%) uterine cancers. Among the incident cancers, 249 (14.2%), 333 (7.3%), 230 (69.3%), 234 (58.9%), 730 (83.1%), and 106 (14.8%) deaths from bladder, breast, esophagus, gastric, pancreatic, and uterine cancer, respectively, were reported.

**Table.** Baseline Characteristics of 139 896 Participants in the Prostate, Lung, Colorectal, and Ovarian Cancer Screening Trial

Characteristic	Participants, No. (%)						
	Total population (n = 139 896)	Cancer diagnosis					
		Bladder (n = 1751)	Breast^a^ (n = 4552)	Esophageal (n = 332)	Gastric (n = 397)	Pancreatic (n = 878)	Uterine^a^ (n = 716)
Age at start of follow-up, y							
65^b^	93 871 (67.1)	930 (53.1)	2810 (61.7)	187 (56.3)	203 (51.1)	490 (55.8)	434 (60.6)
66-69	26 069 (18.6)	457 (26.1)	993 (21.8)	73 (22.0)	94 (23.7)	196 (22.3)	168 (23.5)
70-74	19 947 (14.3)	364 (20.8)	750 (16.5)	72 (21.7)	100 (25.2)	192 (21.9)	114 (15.9)
≥75	9 (<0.1)	0	0	0	0	0	0
Sex							
Male	68 012 (48.6)	1378 (78.7)	NA	275 (82.8)	273 (68.8)	447 (50.9)	NA
Female	71 884 (51.4)	373 (21.3)	4552 (100.0)	57 (17.2)	124 (31.2)	431 (49.1)	716 (100.0)
Race^c^							
Non-Hispanic							
White	123 824 (88.5)	1629 (93.0)	4085 (89.7)	300 (90.4)	304 (76.6)	744 (84.7)	644 (89.9)
Black	6950 (5.0)	44 (2.5)	220 (4.8)	11 (3.3)	36 (9.1)	56 (6.4)	33 (4.6)
Other^d^	9058 (6.5)	77 (4.4)	247 (5.4)	21 (6.3)	57 (14.4)	78 (8.9)	39 (5.5)
Smoking status^e^							
Never	65 502 (46.8)	466 (26.6)	2564 (56.3)	86 (25.9)	146 (36.8)	402 (45.8)	439 (61.3)
Current	14 112 (10.1)	271 (15.5)	334 (7.3)	58 (17.5)	52 (13.0)	115 (13.1)	42 (5.9)
Former	60 263 (43.1)	1014 (58.9)	1653 (36.3)	188 (56.6)	199 (50.1)	361 (41.1)	235 (32.8)
Randomization group							
Intervention	70 531 (50.4)	867 (49.5)	2336 (51.3)	143 (43.1)	202 (50.9)	468 (53.3)	343 (47.9)
Control	69 365 (49.6)	884 (50.5)	2216 (48.7)	189 (56.9)	195 (49.1)	410 (46.7)	373 (52.1)
Reported aspirin use in the last 12 mo^f^							
No	71 665 (51.2)	810 (46.3)	2626 (57.7)	150 (45.2)	212 (53.4)	442 (50.3)	415 (58.0)
Yes	68 231 (48.8)	941 (53.7)	1926 (42.4)	182 (54.8)	185 (46.6)	436 (49.7)	301 (42.0)
Reported aspirin frequency in the last 12 mo, times/wk^f^							
<3	114 511 (81.9)	1400 (80.0)	3782 (83.1)	254 (76.5)	334 (84.1)	713 (81.2)	605 (84.5)
≥3	25 385 (18.2)	351 (20.0)	770 (16.9)	78 (23.5)	63 (15.9)	165 (18.8)	111 (15.5)

Abbreviation: NA, not applicable.

^a^ Only female participants were included in the analyses of breast and uterine cancers.

^b^ Individuals were entered into the current analysis at exactly age 65 years, which may have been some time after trial randomization.

^c^ A total of 64 participants had missing values for race.

^d^ Other included Hispanic, Asian, Pacific Islander, and American Indian.

^e^ A total of 19 participants had missing values from smoking status.

^f^ Reported aspirin use from baseline questionnaire.

### Incident Cancer Analysis

**Figure 2.**
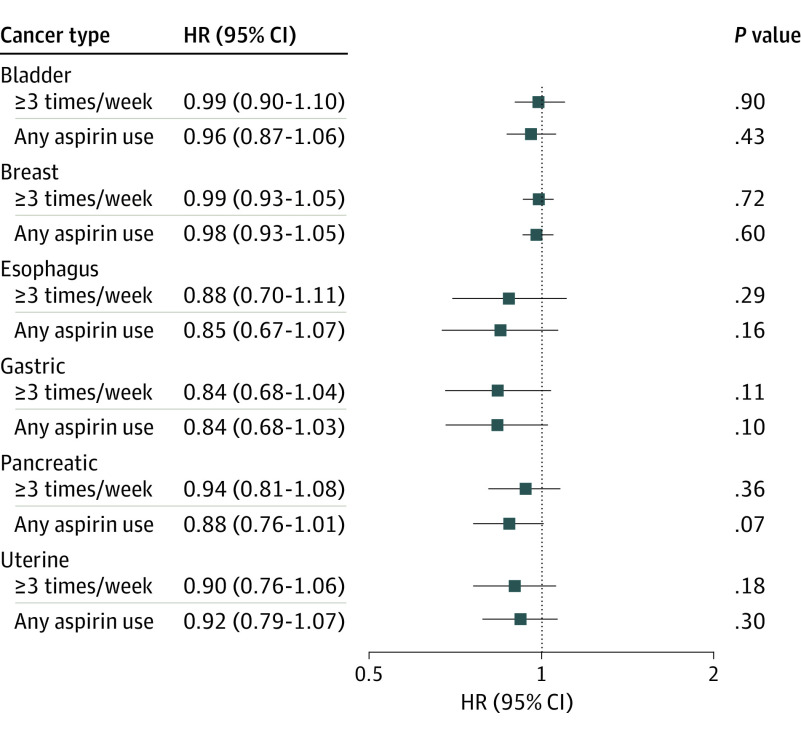
Adjusted Hazard Ratios (HRs) and 95% CIs for Cancer Incidence by Aspirin Use in the Prostate, Lung, Colorectal, and Ovarian Cancer Screening Trial Bladder, esophageal, gastric, and pancreatic cancer models were adjusted for randomization group, sex, race, smoking status, and history of heart attack, stroke, hypertension, and diabetes. Breast and uterine cancer models analyzed only female participants and were adjusted for randomization group, race, smoking status, and history of heart attack, stroke, hypertension, and diabetes.

The results of the Cox proportional hazards models investigating the association between aspirin use and cancer incidence are shown in Figure 2. Aspirin use at least 3 times/week was not associated with risk of bladder (HR, 0.99; 95% CI, 0.90-1.10), breast (HR, 0.99; 95% CI, 0.93-1.05), esophageal (HR, 0.88; 95% CI, 0.70-1.11), gastric (HR, 0.84; 95% CI, 0.68-1.04), pancreatic (HR, 0.94; 95% CI, 0.81-1.08), or uterine (HR, 0.90; 95% CI, 0.76-1.06) cancers. Similarly, when we evaluated any aspirin use, no association with cancer risk was observed (Figure 2). The unadjusted models of cancer incidence and covariates included in the multivariable model are included in eTable 1 and eTable 2 in the Supplement.

### Survival Analysis

**Figure 3.**
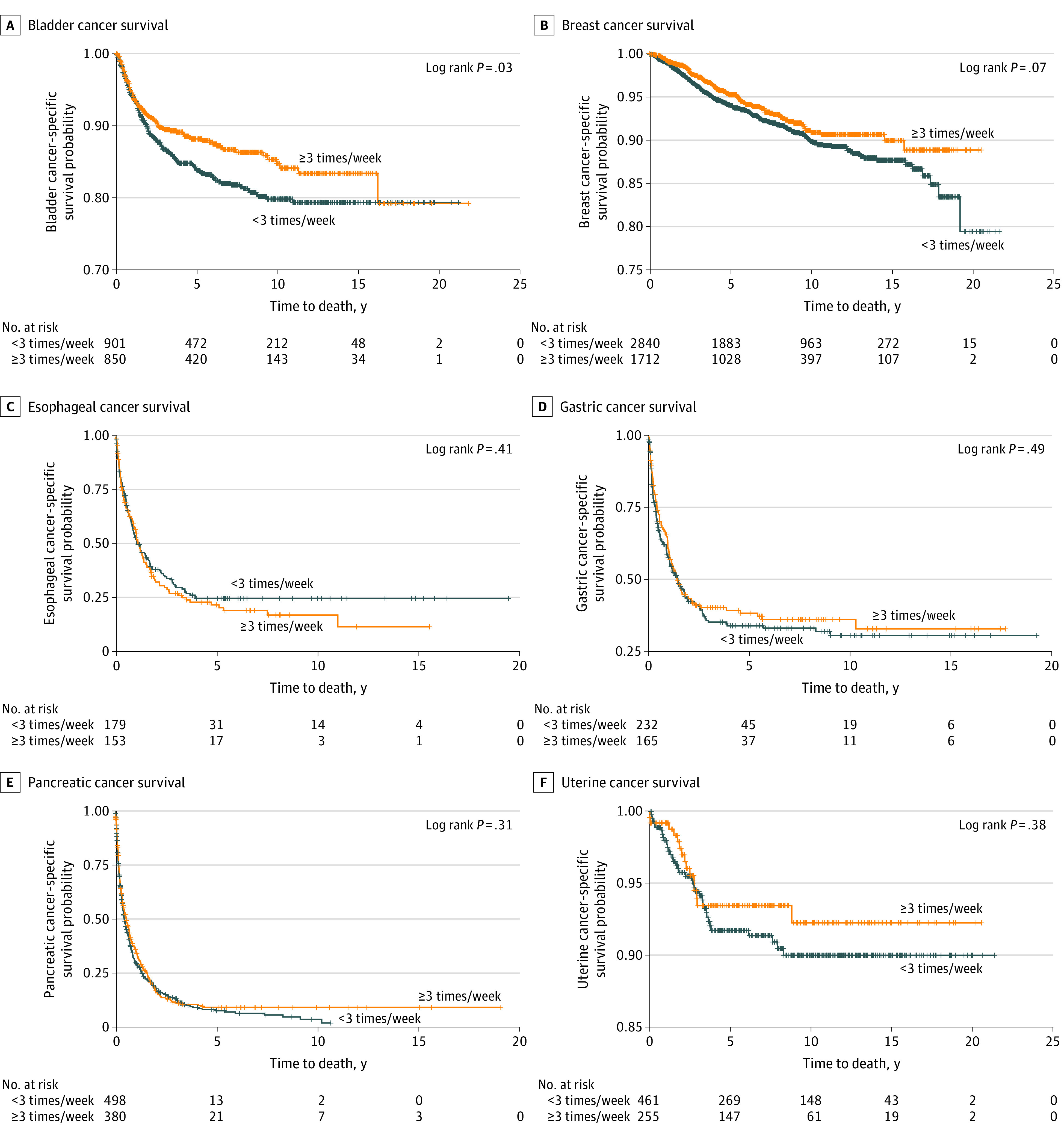
Unadjusted Kaplan-Meier Estimates of Cancer Site–Specific Survival Among Participants in the Prostate, Lung, Colorectal, and Ovarian Cancer Screening Trial Cancer Screening Trial

Figure 3 shows the results of the Kaplan-Meier survival analysis. Participants with bladder cancer had significantly increased survival with aspirin use at least 3 times/week compared with aspirin use less than 3 times/week (log-rank *P* < .001) (Figure 3A). Breast cancer survival with aspirin use at least 3 times/week was not significant (log-rank *P* = .07) (Figure 3B). No survival differences were observed between groups for esophageal, gastric, pancreatic, or uterine cancers (Figure 3C-F).

**Figure 4.**
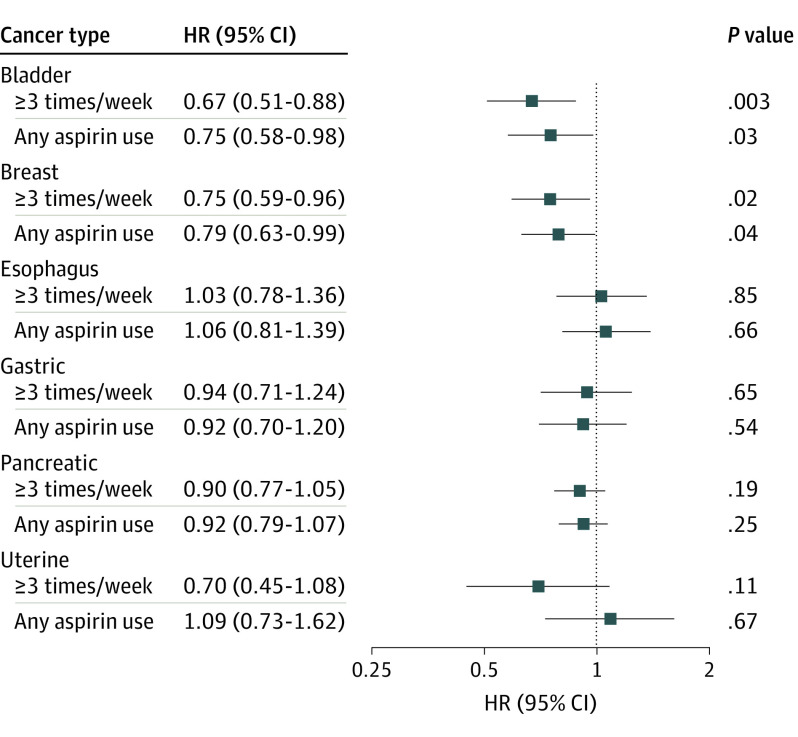
Adjusted Hazard Ratios (HRs) and 95% CIs for Cancer Survival by Aspirin Use in the Prostate, Lung, Colorectal, and Ovarian Cancer Screening Trial Bladder, esophageal, gastric, and pancreatic cancer models were adjusted for age at diagnosis, randomization group, sex, race, smoking status, and history of heart attack, stroke, hypertension, and diabetes. Breast and uterine cancer models analyzed only female participants and were adjusted for age at diagnosis, randomization group, race, smoking status, and history of heart attack, stroke, hypertension, and diabetes.

In the multivariable proportional hazards models, participants who reported aspirin use at least 3 times/week showed improved survival for bladder (HR, 0.67; 95% CI, 0.51-0.88) and breast (HR, 0.75; 95% CI, 0.63-0.99) cancer compared with those who reported aspirin use less than 3 times/week (Figure 4). No association between aspirin use at least 3 times/week and survival was observed for esophageal, gastric, pancreatic, and uterine cancers. Similar to aspirin use at least 3 times/week, any reported aspirin use was associated with reduced risk of death from bladder (HR, 0.75; 95% CI, 0.58-0.98) and breast (HR, 0.79; 95% CI, 0.63-0.99) cancers compared with no reported aspirin use after adjusting for covariates (Figure 4). Also consistent with the findings for aspirin use at least 3 times/week, no association was observed between any reported aspirin use and esophageal, gastric, pancreatic and uterine cancer survival. The unadjusted models of cancer survival and covariates included in the multivariable model are included in eTable 3 and eTable 4 in the Supplement.

## Discussion

In the current study, we evaluated the association between aspirin use and cancer incidence and survival. Specifically, we investigated these associations for bladder, breast, esophageal, gastric, pancreatic, and uterine cancers. We did not observe any significant associations between aspirin use and cancer incidence, although we observed a significant association between aspirin use and bladder and breast cancer survival. Although aspirin use at least 3 times/week was associated with the strongest risk reduction, any aspirin use was associated with increased bladder and breast cancer survival. These results may indicate that for some cancer types, any aspirin use may be advantageous; however, greater benefit may be observed with increased frequency of use. Furthermore, we found that for individuals with several prevailing cancer types (eg, gastric, pancreatic, uterine), aspirin use may not be associated with a similar survival benefit. These results are consistent with additional reports of aspirin use and risk of cancer mortality, such as a recent clinic-based study of patients with bladder cancer that found that daily aspirin use was associated with increased 5-year survival following radical cystectomy, and provide further detail on the association between aspirin use and cancer site–specific survival, therefore lending novelty to our analysis.^32,46^

Of note, our study assessed the association of aspirin use with the risk of bladder, pancreatic, and uterine cancer incidence and survival, which are unexplored avenues. Several hypotheses regarding aspirin’s mechanism of action and its impact on bladder and breast cancer survival have been developed. Compared with normal epithelial cells, RNA and protein expression of cyclooxygenase-2 (COX-2) and urinary prostaglandin E2 is increased in urothelial carcinoma, suggesting upregulation of the COX-2 pathway during cancer progression.^47,48,49,50^ Similarly, elevated expression of COX-2 in breast cancer has been shown to be a predictor of disease outcome (eg, progression, decreased survival).^51,52^ This may be partly due to the mechanistic interplay between angiogenesis, cell proliferation, apoptosis, and inflammatory processes.^53^

A focus of work surrounding the efficacy of aspirin as a cancer preventive agent has occurred in 2 contexts: gastrointestinal cancers (primarily colorectal cancer) and younger individuals. Despite compelling in vitro evidence, population-based studies have not consistently supported a role for aspirin in the prevention of esophageal and gastric cancers.^54,55,56^ Moreover, even though aspirin use has suggested efficacy in the prevention of colorectal cancer incidence and cancer-associated mortality in individuals between the ages of 50 and 69 years, there is a lack of observational and randomized clinical trial data for younger and older individuals. The ASPREE trial showed increased risk of cancer-associated death, including colorectal cancer, in the (low-dose) aspirin group compared with the placebo group, with this effect being particularly pronounced among individuals with late-stage or metastatic disease.^9,10^ Of note, ASPREE study participants were aged 65 years or older, many were cancer survivors, and those with occult disease were not excluded by the exclusion criteria.^9^ In addition, most ASPREE participants were not taking regular low-dose aspirin before starting the trial. ASPREE also showed no significant reduction in the incidence of all solid cancers or of colorectal cancer in the aspirin group.^10^ The protective association of aspirin use with all-cause, all-cancer, and colorectal cancer mortality in older individuals was recently reported, and the current study provides further detail on several major cancers reported in a similar PLCO cohort.^33^

### Strengths and Limitations

There are several significant strengths to this study to note. First, we obtained data regarding aspirin use frequency from a large cohort and during a long follow-up period. Moreover, the extended follow-up period allowed for the collection of supplemental participant data, permitting time-varying analysis. Despite the strengths, there are important limitations to note. The present study is a secondary analysis of a cancer screening trial, and the exposure and outcomes of interest were not the primary designated study outcomes. Therefore, for some of our cancer sites of interest, such as esophageal or uterine cancer, we were limited in the number of cohort deaths. Further, we evaluated cancer incidence and site-specific survival in 6 separate cancers, resulting in 12 independent comparisons; therefore, multiple comparisons were not controlled for in the analysis. Aspirin dose was not included in the analysis because this information was collected as part of the SQ, but not the BQ; however, some analyses suggest that any aspirin use, regardless of dose, may be associated with cancer incidence and mortality.^57^ Furthermore, aspirin use was assessed at baseline and/or follow-up, and only 1 time was used for the survival analysis; however, it is possible that participant aspirin use fluctuated throughout follow-up, which is not accounted for in our models.

## Conclusions

This study did not find associations between aspirin use and bladder, breast, esophageal, gastric, pancreatic, or uterine cancer incidence in the PLCO Cancer Screening Trial. We did find a positive association between any aspirin use and bladder and breast cancer survival, although this was not seen for esophageal, gastric, pancreatic, or uterine cancer survival. The results presented here add to the accumulating evidence that aspirin may improve survival for some cancers. Although prior research has been most heavily concentrated in gastrointestinal cancers, our analysis extends the advantages associated with aspirin use to other cancers, such as bladder and breast cancers. However, although aspirin use may confer a cancer protective effect, it remains necessary to consider the harms, as well as the benefits, of long-term aspirin use.

## Notes

Supplement. **eTable 1. **Unadjusted Hazard Ratios and 95% CIs of Aspirin Use and Risk of Cancer **eTable 2. **Covariates Included in the Multivariable Cox Proportional Hazards of Cancer Risk (Figure 2), Comparing Aspirin Use Less Than 3 Times/Week and at Least 3 Times/Week **eTable 3. **Unadjusted Hazard Ratios and 95% CIs of Aspirin Use and Risk of Cancer-Specific Death **eTable 4. **Covariates Included in the Multivariable Cox Proportional Hazards of Risk of Cancer-Specific Death (Figure 4), Comparing Aspirin Use Less Than 3 Times/Week and at Least 3 Times/Week
