# Supplementary material for: Evaluation of Aspirin Use With Cancer Incidence and Survival Among Older Adults in the Prostate, Lung, Colorectal, and Ovarian Cancer Screening Trial
Source: JAMA Netw Open. 2021 Jan 15;4(1):e2032072. doi: 10.1001/jamanetworkopen.2020.32072 (PMC7811183; doi:10.1001/jamanetworkopen.2020.32072)
Supplement: Supplement. — eTable 1. Unadjusted Hazard Ratios and 95% CIs of Aspirin Use and Risk of Cancer eTable 2. Covariates Included in the Multivariable Cox Proportional Hazards of Cancer Risk (Figure 2), Comparing Aspirin Use Less Than 3 Times/Week and at Least 3 Times/Week eTable 3. Unadjusted Hazard Ratios and 95% CIs of Aspirin Use and Risk of Cancer-Specific Death eTable 4. Covariates Included in the Multivariable Cox Proportional Hazards of Risk of Cancer-Specific Death (Figure 4), Comparing Aspirin Use Less Than 3 Times/Week and at Least 3 Times/Week [file jamanetwopen-e2032072-s001.pdf]

Supplemental Online Content

Loomans-Kropp HA, Pinsky P, Umar A. Evaluation of aspirin use with cancer incidence and survival among older adults in the Prostate, Lung, Colorectal, and Ovarian Cancer Screening Trial. *JAMA Netw Open*. 2021;4(1):e2032072. doi:10.1001/jamanetworkopen.2020.32072

eTable 1. Unadjusted Hazard Ratios and 95% CIs of Aspirin Use and Risk of Cancer

eTable 2. Covariates Included in the Multivariable Cox Proportional Hazards of Cancer Risk (Figure 2), Comparing Aspirin Use Less Than 3 Times/Week and at Least 3 Times/Week

eTable 3. Unadjusted Hazard Ratios and 95% CIs of Aspirin Use and Risk of Cancer-Specific Death

eTable 4. Covariates Included in the Multivariable Cox Proportional Hazards of Risk of Cancer-Specific Death (Figure 4), Comparing Aspirin Use Less Than 3 Times/Week and at Least 3 Times/Week

This supplemental material has been provided by the authors to give readers additional information about their work.

© 2021 Loomans-Kropp HA et al. *JAMA Network Open.*

eTable 1. Unadjusted Hazard Ratios and 95% CIs of Aspirin Use and Risk of Cancer

	Reported aspirin use yes[a]		Reported aspirin use frequency ≥3 times/week[b]	
Cancer site	HR (95% CI)	*p*-value	HR (95% CI)	*p*-value
Bladder cancer	1.18 (1.08, 1.30)	<0.001	1.25 (1.13, 1.37)	<0.001
Breast cancer[c]	1.00 (0.94, 1.06)	0.91	1.00 (0.94, 1.06)	0.92
Esophageal cancer	1.07 (0.86, 1.33)	0.53	1.14 (0.92, 1.42)	0.22
Gastric cancer	0.92 (0.76, 1.13)	0.43	0.95 (0.78, 1.16)	0.62
Pancreatic cancer	0.94 (0.82, 1.07)	0.34	1.00 (0.88, 1.15)	0.97
Uterine cancer[c]	0.95 (0.82, 1.10)	0.49	0.92 (0.79, 1.07)	0.29

[a]Reference: no aspirin use[c]

[b]Reference: <3 times/month

[c]Models for breast and uterine cancer included female participants only

Abbreviations: PLCO = Prostate, Lung, Colorectal, and Ovarian Cancer Screening Trial; HR = hazard ratio; 95% CI = 95% confidence interval

© 2021 Loomans-Kropp HA et al. *JAMA Network Open.*

eTable 2. Covariates Included in the Multivariable Cox Proportional Hazards of Cancer Risk (Figure 2), Comparing Aspirin Use Less Than 3 Times/Week and at Least 3 Times/Week

	Cancer Type					
	Bladder (N=1751)	Breast (N=4552)	Esophageal (N=332)	Gastric (N=397)	Pancreatic (N=878)	Uterine (N=716)
Variable	Adjusted HR (95% CI)	Adjusted HR (95% CI)	Adjusted HR (95% CI)	Adjusted HR (95% CI)	Adjusted HR (95% CI)	Adjusted HR (95% CI)
Sex						
Male	1.0 (ref)		1.0 (ref)	1.0 (ref)	1.0 (ref)	
Female	0.27 (0.24, 0.31)		0.21 (0.16, 0.29)	0.42 (0.33, 0.52)	0.86 (0.74, 0.98)	
Race						
White, non-Hispanic	1.0 (ref)	1.0 (ref)	1.0 (ref)	1.0 (ref)	1.0 (ref)	1.0 (ref)
Black, non-Hispanic	0.54 (0.40, 0.73)	0.87 (0.76, 1.00)	0.62 (0.33, 1.17)	2.19 (1.54, 3.12)	1.31 (1.00, 1.73)	0.77 (0.53, 1.10)
Other	0.57 (0.45, 0.72)	0.86 (0.76, 0.98)	0.80 (0.51, 1.27)	2.17 (1.63, 2.89)	1.27 (1.00, 1.61)	0.84 (0.61, 1.16)
Smoking status						
Never smoker	1.0 (ref)	1.0 (ref)	1.0 (ref)	1.0 (ref)	1.0 (ref)	1.0 (ref)
Current smoker	3.09 (2.64, 3.62)	0.92 (0.82, 1.04)	3.73 (2.62, 5.32)	1.75 (1.25, 2.46)	1.90 (1.54, 2.36)	0.68 (0.48, 0.95)
Former smoker	1.90 (1.70, 2.13)	1.12 (1.05, 1.26)	1.93 (1.47, 2.52)	1.31 (1.05, 1.63)	0.97 (0.84, 1.12)	0.91 (0.77, 1.06)
Randomization arm						
Intervention	1.0 (ref)	1.0 (ref)	1.0 (ref)	1.0 (ref)	1.0 (ref)	1.0 (ref)
Control	1.04 (0.95, 1.14)	0.96 (0.93, 1.05)	1.34 (1.08, 1.67)	1.01 (0.83, 1.23)	0.90 (0.79, 1.03)	1.10 (0.95, 1.28)
History of heart attack						
No	1.0 (ref)	1.0 (ref)	1.0 (ref)	1.0 (ref)	1.0 (ref)	1.0 (ref)
Yes	1.16 (1.01, 1.35)	0.98 (0.85, 1.13)	1.28 (0.93, 1.76)	1.19 (0.87, 1.62)	1.26 (1.01, 1.56)	0.99 (0.69, 1.40)
History of stroke						
No	1.0 (ref)	1.0 (ref)	1.0 (ref)	1.0 (ref)	1.0 (ref)	1.0 (ref)
Yes	1.18 (0.95, 1.48)	0.96 (0.81, 1.15)	0.83 (0.46, 1.49)	1.40 (0.90, 2.16)	0.92 (0.65, 1.31)	0.82 (0.51, 1.32)
History of hypertension						
No	1.0 (ref)	1.0 (ref)	1.0 (ref)	1.0 (ref)	1.0 (ref)	1.0 (ref)
Yes	1.04 (0.94, 1.15)	1.06 (1.00, 1.13)	1.41 (1.13, 1.78)	1.12 (0.91, 1.37)	1.16 (1.01, 1.33)	1.08 (0.92, 1.26)
History of diabetes						
No	1.0 (ref)	1.0 (ref)	1.0 (ref)	1.0 (ref)	1.0 (ref)	1.0 (ref)
Yes	1.30 (1.13, 1.50)	1.11 (1.00, 1.23)	1.15 (0.83, 1.60)	1.64 (1.25, 2.14)	1.70 (1.41, 2.04)	1.62 (1.28, 2.05)

© 2021 Loomans-Kropp HA et al. *JAMA Network Open.*

eTable 3. Unadjusted Hazard Ratios and 95% CIs of Aspirin Use and Risk of Cancer-Specific Death

	Reported aspirin use yes[a]		Reported aspirin use frequency ≥3 times/week[b]	
Cancer site	HR (95% CI)	*p*-value	HR (95% CI)	*p*-value
Bladder cancer	0.80 (0.63, 1.03)	0.08	0.76 (0.59, 0.98)	0.03
Breast cancer[c]	0.83 (0.67,1.03)	0.09	0.80 (0.64, 1.02)	0.07
Esophageal cancer	1.12 (0.86, 1.46)	0.39	1.11 (0.86, 1.44)	0.42
Gastric cancer	0.96 (0.74, 1.24)	0.77	0.91 (0.70, 1.18)	0.49
Pancreatic cancer	0.93 (0.80, 1.07)	0.29	0.93 (0.80, 1.07)	0.31
Uterine cancer[c]	1.20 (0.82, 1.76)	0.35	0.86 (0.57, 1.30)	0.48

[a]Reference: no aspirin use

[b]Reference: <3 times/month

[c]Models for breast and uterine cancer included female participants only

Abbreviations: PLCO = Prostate, Lung, Colorectal, and Ovarian Cancer Screening Trial; HR = hazard ratio; 95% CI = 95% confidence interval

© 2021 Loomans-Kropp HA et al. *JAMA Network Open.*

eTable 4. Covariates Included in the Multivariable Cox Proportional Hazards of Risk of Cancer-Specific Death (Figure 4), Comparing Aspirin Use Less Than 3 Times/Week and at Least 3 Times/Week

	Cancer Type					
	Bladder (N=249)	Breast (N=333)	Esophageal (N=230)	Gastric (N=234)	Pancreatic (N=730)	Uterine (N=106)
Variable	Adjusted HR (95% CI)	Adjusted HR (95% CI)	Adjusted HR (95% CI)	Adjusted HR (95% CI)	Adjusted HR (95% CI)	Adjusted HR (95% CI)
Age at diagnosis	1.06 (1.04, 1.08)	1.04 (1.02, 1.05)	1.03 (0.79, 1.36)	1.02 (1.00, 1.04)	1.03 (1.01, 1.04)	1.03 (1.00, 1.05)
Sex						
Male	1.0 (ref)		1.0 (ref)	1.0 (ref)	1.0 (ref)	
Female	0.88 (0.64, 1.22)		0.90 (0.63, 1.28)	0.99 (0.74, 1.32)	0.94 (0.81, 1.09)	
Race						
White, non-Hispanic	1.0 (ref)	1.0 (ref)	1.0 (ref)	1.0 (ref)	1.0 (ref)	1.0 (ref)
Black, non-Hispanic	1.46 (0.71, 2.99)	1.89 (1.30, 2.75)	0.96 (0.48, 1.92)	0.61 (0.37, 1.00)	0.87 (0.63, 1.19)	1.1 (0.82, 3.57)
Other	0.76 (0.39, 1.48)	0.67 (0.39, 1.15)	1.13 (0.68, 1.89)	0.58 (0.39, 0.86)	1.07 (0.83, 1.38)	0.32 (0.08, 1.31)
Smoking status						
Never smoker	1.0 (ref)	1.0 (ref)	1.0 (ref)	1.0 (ref)	1.0 (ref)	1.0 (ref)
Current smoker	1.38 (0.67, 2.83)	1.77 (1.19, 2.64)	0.94 (0.41, 2.17)	1.95 (0.92, 4.13)	1.24 (0.97, 1.57)	0.89 (0.32, 2.48)
Former smoker	0.65 (0.33, 1.27)	1.17 (0.93, 1.47)	0.84 (0.52, 1.38)	0.92 (0.58, 1.45)	0.94 (0.80, 1.10)	0.90 (0.59, 1.37)
Randomization arm						
Intervention	1.0 (ref)	1.0 (ref)	1.0 (ref)	1.0 (ref)	1.0 (ref)	1.0 (ref)
Control	1.05 (0.82, 1.34)	1.02 (00.82, 1.27)	1.15 (0.88, 1.50)	0.84 (0.65, 1.10)	1.04 (0.89, 1.20)	1.18 (0.80, 1.73)
History of heart attack						
No	1.0 (ref)	1.0 (ref)	1.0 (ref)	1.0 (ref)	1.0 (ref)	1.0 (ref)
Yes	1.38 (0.97, 1.96)	1.24 (0.79, 1.95)	1.22 (0.84, 1.76)	1.18 (0.79, 1.76)	1.12 (0.87, 1.42)	1.16 (0.48, 2.78)
History of stroke						
No	1.0 (ref)	1.0 (ref)	1.0 (ref)	1.0 (ref)	1.0 (ref)	1.0 (ref)
Yes	1.21 (0.71, 2.07)	1.10 (0.60, 2.03)	1.89 (1.01, 3.54)	0.95 (0.53, 1.71)	1.15 (0.78, 1.68)	0.87 (0.27, 2.79)
History of hypertension						
No	1.0 (ref)	1.0 (ref)	1.0 (ref)	1.0 (ref)	1.0 (ref)	1.0 (ref)
Yes	1.08 (0.83, 1.40)	1.01 (0.81, 1.27)	1.08 (2, 1.42)	1.20 (0.92, 1.58)	1.02 (0.88, 1.19)	1.54 (1.02, 2.33)
History of diabetes						
No	1.0 (ref)	1.0 (ref)	1.0 (ref)	1.0 (ref)	1.0 (ref)	1.0 (ref)
Yes	0.99 (0.68, 1.44)	1.42 (0.99, 2.03)	1.20 (0.81, 1.78)	1.04 (0.72, 1.50)	1.11 (0.91, 1.36)	1.22 (70, 2.13)

© 2021 Loomans-Kropp HA et al. *JAMA Network Open.*
